# Integrating the HFACS Framework and Fuzzy Cognitive Mapping for In-Flight Startle Causality Analysis

**DOI:** 10.3390/s22031068

**Published:** 2022-01-29

**Authors:** Abiodun Brimmo Yusuf, Ah-Lian Kor, Hissam Tawfik

**Affiliations:** 1 School of Built Environment, Engineering and Computing, Leeds Beckett University, Leeds LS6 3QS, UK; A.Kor@leedsbeckett.ac.uk (A.-L.K.); htawfik@sharjah.ac.ae (H.T.); 2 College of Engineering, University of Sharjah, Sharjah 27272, United Arab Emirates

**Keywords:** flight simulation, human factors, flight safety, training, loss of control, startle, situation awareness, fuzzy cognitive maps

## Abstract

This paper discusses the challenge of modeling in-flight startle causality as a precursor to enabling the development of suitable mitigating flight training paradigms. The article presents an overview of aviation human factors and their depiction in fuzzy cognitive maps (FCMs), based on the Human Factors Analysis and Classification System (HFACS) framework. The approach exemplifies system modeling with agents (causal factors), which showcase the problem space’s characteristics as fuzzy cognitive map elements (concepts). The FCM prototype enables four essential functions: explanatory, predictive, reflective, and strategic. This utility of fuzzy cognitive maps is due to their flexibility, objective representation, and effectiveness at capturing a broad understanding of a highly dynamic construct. Such dynamism is true of in-flight startle causality. On the other hand, FCMs can help to highlight potential distortions and limitations of use case representation to enhance future flight training paradigms.

## 1. Introduction

Aviation human factor concepts, and their relationship to an in-flight startle, are investigated in this paper by establishing a hierarchy of key drivers of such startle reactions and exploring the possible connections between them. Furthermore, this paper aims to evidence and demonstrate the use of fuzzy cognitive maps (FCM) to analyze in-flight startle causality objectively.

We proceed on the premise that decision-making during in-flight operations involving fast-evolving challenges can be significantly tasking, cognitively [1,2,3]. However, despite normal safe flight conditions requiring constant monitoring, when an unexpected event occurs in flight, resulting in an aircraft upset, existing research has reported a loss of control (LOC) as an imminent consequence of this situation [4,5,6]. Moreover, in the past decade, LOC-related incidents have contributed to a significant level of all fatal incidents [7,8,9,10]. Thus, currently, LOC assumes a critical focal point for aviation safety improvement.

Other studies have suggested that startle effects impact pilot performance and lead to an LOC [2,11]. Undeniably, the impact of startle on LOC situations is that decision-making is significantly degraded, especially in unexpected or unforeseen circumstances that trigger startle responses by pilots in control and monitoring. The study of attention-related human performance-limiting states (AHPLSs) is significant in current research [12,13]. The issue of startle-potentiated loss of control is a crucial element of these performance-limiting states and affects even highly experienced pilots with devastating consequences [2,14].

Consequently, we agree with the view that pilot training improvements must be a critical part of the industry-wide strategy to mitigate LOC-related incidents [7,8,9,12]. A crucial component of this mitigation relies on modern flight simulation technologies, providing high fidelity and quality training programs to equip pilots with the necessary flying skills that are transferrable to real-life operations. Therefore, understanding the pertinent issues around LOC is key to developing appropriate training protocols.

### 1.1. Research Aim and Objectives

This study aims to develop a view on startle causality as it might affect a pilot during in-flight operations through fuzzy cognitive maps of human factors concepts. The following list of research objectives will support this aim: (i) evaluate dynamic causal variables that act as barriers to pilots’ optimal responses to a startling event; (ii) develop fuzzy cognitive maps to demonstrate the efficacy and practicality of FCMs to prototype causal pathways to startle events rapidly; and (iii) provide a summary report of the mapping outputs discovered as a precursor to further investigation.

### 1.2. Rationale

Most efforts in the relevant literature have focused on the commercial and transport aviation sectors and the associated ancillary operations. This disparity in coverage has provided the impetus to cater to the general aviation (GA) sector [7,15,16]. Therefore, this paper discusses research that focuses on benefitting GA operations but applying methods applicable across the board. A preliminary discussion of this research is available in [17].

Principally, the gap addressed is within the general aviation domain. The relevance of this work stems from the view that GA (Part 91) operations are significantly underrepresented in the existing discourse on LOC research, as highlighted by [5,15,16,18].

Another compelling reason for this work is that human factors continue to plague human endeavor, especially in critical situations, such as flying an aircraft. The very human issue of losing situational awareness and startle (which occurs at the first level of the situational awareness model) can affect pilots of all levels [19,20,21,22]. This paper outlines a process to assess in-flight startle propagation—causality, with a human in the loop perspective. Invariably, this supports the eventual development of mitigative training protocols in the flight simulator training paradigm. The HFACS framework helps us to build a perspective on in-flight startle. However, firstly, it is essential to expound on the startle concept. Some experts view it as an emotional reaction of humans (note that this is debatable because others view startle as a reflex reaction) from a psychophysiological perspective that transcends all pilot operations’ categories and experiences. These perspectives have pros and cons, with consequences for application design and development. In this study, we consider the susceptibility of a pilot to startle given the presence of human factors and therefore adopt the definition of startle from Rivera et al. as follows: *“In aviation, the startle effect can be defined as an uncontrollable, automatic reflex that is elicited by exposure to a sudden, intense event that violates a pilot’s expectations”* [22].

The notion of a “startle” (in terms of pilot performances under pressure/stress) is also strongly related to the situational awareness (SA) construct [2,12,14,22], and other factors, such as the interconnectivity of the pilot’s mental model about the aircraft state, influenced by environmental factors. Research on situation awareness is covered in [19,21,23,24] amongst others but is beyond the scope of this study. For context, however, the widely accepted situational awareness (SA) model comprises three primary levels: perception (level 1), comprehension (level 2), and projection of future state status (level 3) [21]. Based on [20,21,23], this paper adopts the view that startles would be prevalent at the level 1 (perception) stage of SA. Therefore, it is plausible that at that level of awareness, a fast appraisal of a situation (constrained by the fuzzy nature of a “knee jerk” emotive response to an unexpected stimulus) would most likely influence the decision-making, with the potential emergence of LOC.

### 1.3. Contribution and Organization of Paper

This paper adopts the view that by exploring and formulating ideas on startle and its impacts on performance, we can interrogate important human factors affecting pilot decision-making in an emergency. The widely reported human factors analysis and classification system (HFACS) [25] provides a sound foundation for this purpose. To this end, Section 2 offers pertinent discussion on relevant work related to the modeling of human–machine interactions. We highlight that modeling cognitive-based behaviors in operational settings and the notion of visual attentional resource allocation are undeniably, closely intertwined with the decision-making process.

Section 3 discusses the adopted methodology, which includes a summary of the fuzzy cognitive map as a construct and its relevance for the study of startle’s impact on pilot performance. We suggest that the FCM (used for human factors distillation and objective hierarchy assignment) provides an approximately factual representation. Next, we outline the underlying principles and application of FCMs for startle analysis based on human factor concepts. Section 4 summarizes how the FCM affords a mechanism for evaluating human functional factors in completing a flight task, which may eventually impact the pilot’s reaction to an unexpected and startling event. Finally, Section 5 presents a summary and recommendations for future work.

## 2. Related Work

Research literature [26,27,28,29] forms the foundational basis for our reasonings on situation awareness modeling, visual attention allocation, cognitive modeling, and human factors-related engineering. These are all fundamentally related to the effectiveness of a pilot’s decision-making, safety limitations, and time constraints in an ensuing emergency.

The HFACS framework [25] has been deployed in various disciplines [30,31,32,33,34] and applied extensively due to its encompassing yet customizable applicability. However, in line with the present context, it is used to unravel insights into startle causality. The application seeks to embed simulated flights with appropriately chosen unexpected events to provide pilots with startle resilience training. Here, startle causality is viewed through the lens of an uncertainty model of the cause-and-effect conundrum on how human factors may be connected. Furthermore, we consider that the uncertainty and unpredictability of domain elements (human factors) interactions results in a “fuzzy” representation reflecting the modality and abruptness of an unexpected evolving in-flight situation capable of causing startle.

Several tools are available to support our understanding in this domain. Examples include Man-Machine Integration Design and Analysis System MIDAS [35,36,37,38] and Integrated Safety Assessment Model (ISAM). Of pertinent interest to this article, [3] employed ISAM to evaluate general aviation safety in the National Airspace System (NAS) within an unsuccessful aircraft maneuvering context, resulting in loss of control. The ISAM utilizes event sequence diagrams (ESDs) with fault trees that depict relevant parameters and is a well-known and documented causal risk model. Svensson in [39] also describes an episodic analysis method applied to the study of air traffic controllers and their situation awareness across tasks of various complexities. However, this method also relied on the implementation of specific associated technology to make sense of the pertinent constraints in its domain - in this case, eye tracking outputs were used to make such analysis. This provides good inspiration for further considerations when testing is done in the future. Graham [40] also presents and discusses the use of ISAM as a tool to investigate and find mitigative solutions regarding runway safety operations. Additionally, the FAA national runway safety report of 2015 [41] also implements the ISAM to determine risk baseline measures and forecast safety impacts of changes implemented.

The Adaptive Control of Thought-Rational (ACT-R) framework [23] is another abstraction theorizing human cognition based on psychological experiments. Principally, this cognitive analysis method seeks to develop a model-based implementation of algorithms (based on general assumptions of human understanding and the domain of interest). This process is known as knowledge engineering. Models developed can subsequently be deployed for conducting a comparative analysis of actual tasks (assessed based on traditional measures of cognitive psychology). These performance measures encompass task accuracy, performance completion time, and neurological measures, such as MRI outputs (note that these measures are beyond this study’s scope). However, the entire knowledge engineering process aims to provide insight into how humans recall information and how this information recall process supports problem-solving. However, albeit quite successful in its use [16,32], this framework is only considered for its inspirational value to this research.

The ACT-R framework is rather instructive and complementary in philosophy to the chosen FCM method, which affords an in-depth understanding of startle causal factors and organizing these factors into a hierarchy. Another reason for using FCM is its potential to efficiently capture a startle causality’s theoretical and practical aspects. Additionally, the FCM Expert tool developed by [42] permits the application of machine learning (ML) algorithms to express creative decision-making, where a more vague understanding dominates the subject of interest.

Equation (1) describes the main elements at play within the SEEV framework from [19,27], applied to piloting an aircraft and relevant to this research. The model is pertinent because it provides a succinct and rational overview of how visual attendance to a task in a modern cockpit relies on resource allocation and the available bandwidth. Firstly, it gives a frame of reference for considering visual acuity limitations, in the context of disrupted scanning activity by the pilot, due to being startled. Secondly, it provides insight into the dynamism of in-flight circumstances related to goal attainment or ongoing flight management task execution. According to [19,27,43], attention resource allocation is described by the following equation:

P = a − b + c + d(1)

where:

- P represents the pilot’s level of visual attendance to a problem inflight.
- “a” is the salience component representing how swiftly the pilot captures the onset of the event.
- “b” is the effort factor required to move attention around and across the relevant critical information or area of interest (AOI) in the cockpit environment. This notion of effort has great significance in the ergonomics and overall effectiveness of the cockpit as an environment involving concurrent cognitive activity in an emergency.
- “c” is a value assigned to expectancy—noted as the probability cueing of an event. Of course, this has a significant implication for understanding spatial attention while being intensely focused on a very dynamic situation, such as an airplane upset.
- “d” is simply the value placed on a task in the context of an event, such as unexpected but violent clear air turbulence (CAT).

**Figure 1:**
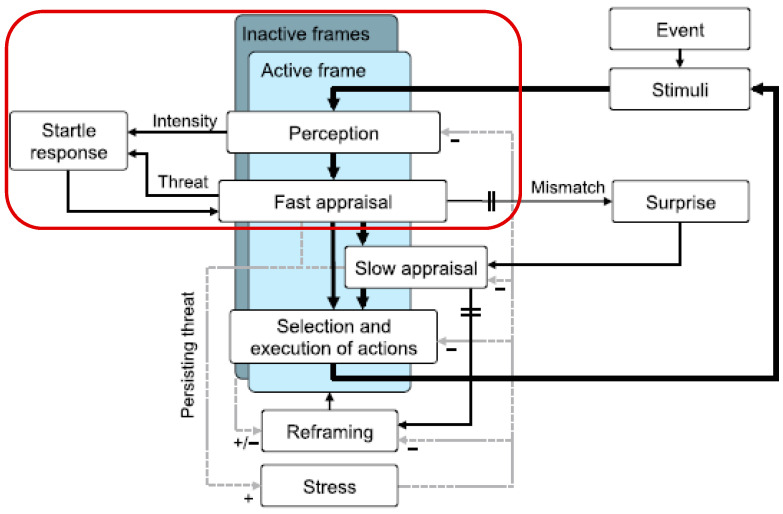
Startle (red boundary) pathway relative to surprise. Adapted with permission from Landman et al., 2017. Copyright 2017 Human Factors.

Considered in combination with the SEEV framework, Figure 1 shows the startle and surprise pathways developed by Landman et al. [14], which we find instructive. The highlighted (red boundary) path to a startle response forms the basis for the following discussion. The proposed model examines the nature of surprise and startle from a system’s perspective and considers the highly dynamic nature of inflight tasks, thus laying the foundation for evaluating the effects of in-flight startle and surprise responses. With the above-mentioned in mind, we can develop a summary conceptualization of the startle process path for a use case, considering only the Landman model’s fast appraisal and perception pathway, leading to activation of the startle reflex.

We can also consider a future state where effort becomes ingrained in the pilot, typically when they are at what can be called an expert level and are very familiar with their operational environments, the cockpit of the aircraft class/type/configuration. If exposure to specific events become routine, then the salience element is also diminished to the extent that an optimized model can be obtained that considers attention allocation only based on the expectancy and value entities multiplicatively. The SEEV model provides a probabilistic estimation of how attending to some point of interest P(AOI) is governed by the influence of perception filters in a larger AOI. A linear weighted combination of the four concepts captures this probability of attendance (in practical terms). Equation (2) from [19] provides a more intuitive form to capture salience (S), effort (EF), expectancy (EX), and value (V), and their respective scaling factors (in lower cases) as shown in the following equation:

P(AOI) = s ∗ S − ef ∗ EF + ex ∗ EX + v ∗ V(2)

The SEEV model (applied to determine visual attention using eye-tracking) seems to produce better accuracy and consistency with actual human behavior [44,45]. Compared to probabilistic scan behavior methods (for predicting the scan pattern given an environmental context), the SEEV model outperforms far more favorably. It highlights the challenge of attending to an unexpected evolving situation and the constraints of expectancy on the choice conundrum of such a scenario or situation.

Notably, this model suggests that cognitive processes exist in parallel (i.e., co-exist) though their channels and required resources are different. For example, a pilot can read (scan) cockpit instruments amidst ongoing tactile feedback processing and consolidation of other auditory instructions. This notion is particularly relevant to this study if we are to conceptualize the potential impact on the pilot’s reactionary task execution ability, which we equate to startle resilience within the first level state of the SA construct. The SEEV model also provides a view of an optimized form as a sum of the expectancy element and the value placed on the relevance, suggesting that given a full calibration of a task/event (simulated training, for example), this interaction could lead to reduced effort to achieve attendance to the ensuing problem. A full consideration of this framework is beyond the scope of this work. However, assurances can be obtained from [27,37,43,44]. Our study primarily focuses on its applicability in human–machine interaction, where the effectiveness of workspace environment scanning, and attention constraints are critically important. The cockpit is one such environment where visual scanning and effective information assessment are crucial to success. The visual comprehension of available information by pilots in a highly stressed situation could be examined objectively, based on the SEEV model, assuming we could obtain a normalized weighting of the human factors’ variables (causal inputs).

**Figure 2:**
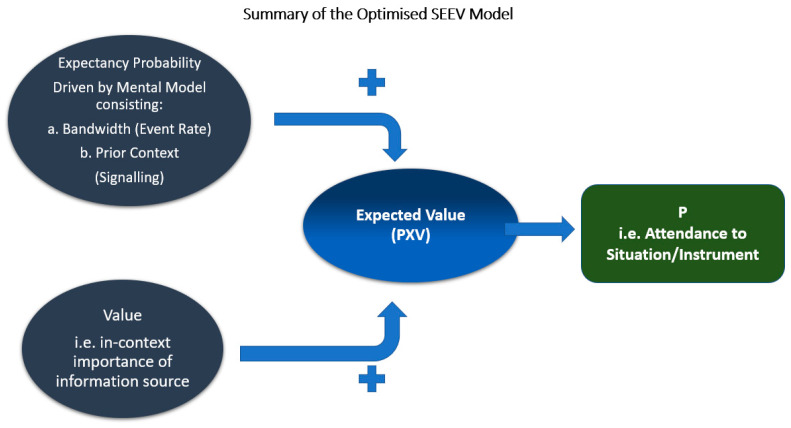
An overview of the optimized SEEV framework representation. Adapted with permission from [Wickens], 2003 Wickens et al.

Figure 2 depicts an optimized structure of the SEEV system as per [19,46] for this purpose. This representation assesses the allocation of visual attention resources during the recommended Aviate, Separate, Navigate, and Communicate ADM process, which pilots use in an emergency. It also represents an optimal expectancy model based on expert pilots’ decision-making.

This structure is valuable for inferential analyses. It supports reflection on the “what-if” scenarios when designing befitting situations of strained visual attention to understand how a pilot may react in unexpected circumstances. Additionally, it also guides reflection on how the degradation of visual attention aligns with the startle effect modality and the potential impact on a pilot’s task performance.

## 3. Fuzzy Cognitive Maps and Modeling Startle

The main goal of an FCM is to model causal knowledge [47,48,49,50]. FCMs are a digraph representation consisting of concept nodes and causal edges with weights that depict the strength of the relationships between the nodes. The FCM method, in this paper, shows its usefulness for four essential functions. It is explanatory, predictive, reflective, and strategic. The predictive function predicts future actions and tendencies that a system agent (node) would contribute to outcomes. In our case, we attempt to predict the concept(s) that presents the highest risk for in-flight startle with the FCM convergence plots. This prediction function supports real-world modeling and subsequent analysis of any experiment results collated from the context of the converged outcomes. Additionally, the reflective function of an FCM provides a means of assessing the adequacy of a decision profile, given the input of system actors influencing the domain of interest.

Furthermore, a robust FCM also provides an enabling strategic function. It lends itself to generating prototypes of expert knowledge descriptions, of a complex dynamic scenario, and the interaction of an operator’s highly active human (psychophysiological) response. Qualitatively, the FCM provides a flexible, robust, and objective explanatory representation of the domain under review and any potential distortions or limitations of the use case representation.

The following sections discuss the FCM’s underlying theories, and the modeling process used for this research.

### 3.1. Underlying Concepts: Fuzzy Sets, Startle Propagation, and FCMs

Fuzzy cognitive mapping is a technique developed by Kosko in 1986 as an extension of cognitive maps. It uses a fuzzy logic viewpoint to model causal knowledge [48]. The FCM creates a directed graph that depicts concepts (nodes) and causal edges pertinent to the domain. In this directed graph representation, the fuzzy weights of any related concepts in the map rely on the relationship strength between nodes displayed as edges. Conventional logic typically represents the output of a variable as a binary true (1) or false (0) output state.

**Figure 3:**
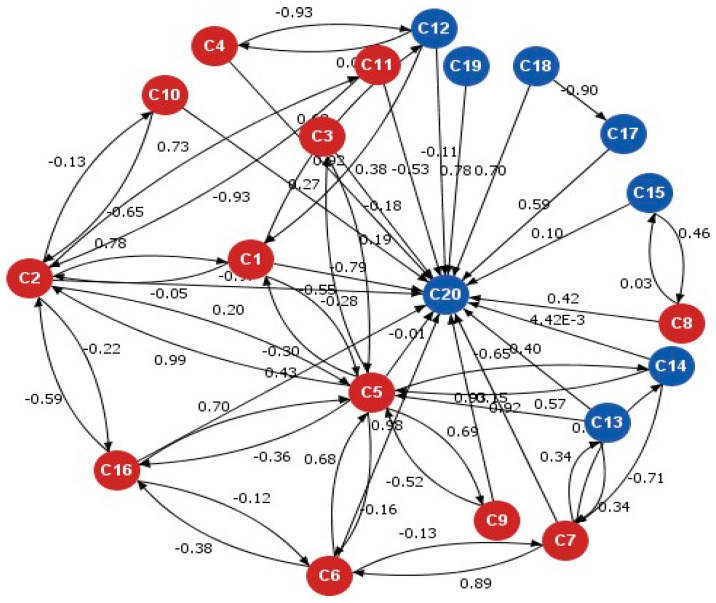
A fuzzy cognitive map demonstrating the notional causal relationships. Twelve nodes (red) are decision drivers, feeding into C20, the startle concept.

On the other hand, fuzzy logic represents the value of such a variable in the continuum from 0 to 1. For instance, an expert panel member determining how impactful a causal variable is for startle might ascribe a weight of 0.25 or 0.87. This value translates to a view of partially true or false (i.e., in terms of being impactful to the elicitation of startle). The ascribed value provides intuitive regard for the relationship strengths amongst concepts in the FCM, as depicted in Figure 3. Using fuzzy sets, we can demonstrate the mathematical construct of fuzzy logic. This logic suggests that, in a crisp set of values, membership or non-membership of an element, say ‘X, in a set *A* is described by a characteristic function as follows:

*µ_A_*(*x*), where *µ_A_*(*x*) = 1 if *x* ∈ *A* and *µ_A_*(*x*) = 0 if *x* ∉ *A*.

The fuzzy set theory extends this concept by suggesting the notion of a defined partial membership. This partial membership concept means that a fuzzy set *A* on a notional universe U is characterized by a membership function of an element with values in the interval [0, 1]. In essence, this set admits all uncertainties associated with the variable with a graded membership [51,52]. For the FCM reasoning process, a simple mathematical formulation is used. Values of the concept C*_i_* at a point in time *t* are denoted by the state vector *A_i_*^(*k*)^ (see Equation (1) below):

*A*^(*k*)^ = [*A_i_*^(*k*)^, … *A_n_*^(*k*)^] (3)

The state vector representation is a point within a fuzzy hypercube 1*^n^* = [0, 1]*^n^* that suggests system behavior at a point in multidimensional space [51,53]. The hypercube epitomizes a system with an input vector *A*^(0)^ within the multidimensional space of concepts. The system hypercube 1*^n^*, once activated, gradually converges to either an equilibrium/stable, chaotic point, or a periodic attractor within the hypercube. The periodic attractor depends on the system’s input vector(s) value.

A fuzzy digraph may structurally exemplify a fuzzy cognitive map with feedback (see Figure 3).

In this form, it is analogous to a collection of neural processing units and weighted relations (which could be positive, negative, or neutral), signifying the levels of causality [54]. To reiterate, the FCM is a system representation that expediently depicts concepts (i.e., variables of the system) and weighted causal relations amongst these concepts. Each concept is characterized by its activation degree (initial weight determined from experts’ input), which denotes to what extent a variable is considered dominant (or otherwise) in the system. Three possible types of causal relationships between concepts *C_i_* and *C_j_* express the influence of one concept on another (i.e., interaction between or amongst variables) as follows:

- *w_ij_* > 0 indicates a positive causality, then an increase (decrease) in *C_i_* will increment the effect concept *C_j_* with intensity |*w_ij_*|.
- *w_ij_* < 0 means a negative causality, then an increase (decrease) in *C_i_* will reduce the effect concept *C_j_* with intensity |*w_ij_*|.
- *w_ij_* = 0 denotes the absence of a causal (or, in other words, neutral) relationship between concepts *C_i_* and *C_j_*.

Human factors in this study, forming the list of causal variables for developing the domain representation, were adapted from the HFACS taxonomy on human factors established by [25]. In their work, they provide dimensions to consider human factor errors, including ergonomic, behavioral, aeromedical, psychosocial, and Organizational perspectives. These perspectives subsequently cascade into four groupings of causal factors: acts and omissions, preconditions and local factors, supervision and local management, and organizational influences, which are further partitioned into 19 concepts for the mapping process. This taxonomy provides a contextual guide to help comprehend the symbiotic structure of our human–aircraft interaction system in terms of what might affect startle resilience given an unexpected evolving event.

Table 1 provides a contextual summary of how a startling event may play out operationally, using the phenomenon of “clear-air turbulence” as an example. Crucially, this conceptualization considers how the ADM process based on [55] would be affected in such a case. Consequently, the staging enables the development of experimental/training protocols to attempt the creation and stimulation of a fast response in the active mental frame of the pilot, sufficient to trigger a startle. Clear air turbulence (CAT) is defined according to [56] as turbulent or bumpy in-flight conditions “in the free atmosphere of interest that is not in, or adjacent to visible convective activity”. In addition, FAA Advisory Circular AC 00-30C [57] define CAT as “sudden severe turbulence occurring in cloudless regions that causes violent buffeting of aircraft”.

The problem with CAT is the difficulty of its detection and measurement and this has been known in a few cases to impact in-flight operations adversely [56,57,58]. Currently, the reporting and awareness of possible “hotspots” varies widely across the globe, creating another potential barrier to accurate information about a future flight in planning wherein a pilot might miss such crucial details. On the issue of CAT and its potential impact on ADM, we frame the conceptual summary around the mechanisms behind the abrupt response pathways that a pilot might adopt in an unexpected stressor event. Consider the process path outlined in [2] on the mode of information gathering through the visual cortex in a very non-routine situation of emergency stress, with very low expectation and self-efficacy. These situations can trigger emotional response pathways in the brain that diminish processing in the frontal cortex, where complex perceptual information is filtered and organized in the brain for reasoning [59] and are in tension with vestibular comprehension [60]. The summation then becomes that abrupt responses by the pilot are driven by activation of both the sympathetic nervous and adrenal-cortical systems, forcing hormones into the bloodstream, and a fight/flight response is brought about.

The elucidation of this emotional response is significant because current technology can easily access some of the physiological manifestations known to be associated with it. Researchers and developers can capture typical effects of the dynamic response, such as pupil dilation and increased heart rates, with eye trackers and other non-intrusive wearable devices, respectively. Concepts about naturalistic decision-making (NDM), as outlined by [61,62,63,64], also highlight the pertinent evolution of cues (visual or auditory, for example), which then drive situation assessment and hypothesis generation, risk assessment, and then through to implementation of an action or series of steps. Within the ground-based training and operational flight paradigms, the ideas mentioned above may also become compounded by the SEEV model discussed earlier; as a result, the convolution of these aspects has a resultant and significant bearing on the eventual outcome action(s) that the pilot implements in response. Thus, we develop the high-level conceptualization of a path to startled behavior using the notion of CAT as an inherent unexpected phenomenon in concert with human factor constraints. The event sequence relies on the reasoning that such a situation can impact all pilot experience and exposure levels, from novice to expert groups. Furthermore, the adoption of a helpful event sequence, as in Table 1, for a CAT-centered event assumes a perfectly working aircraft with the potential failure point being the human element being startled due to the abruptness of the phenomena. As summarized, the event sequence also provides a blueprint for creating such a situation within the flight simulation environment, making it a valid candidate to be considered by training providers in developing the appropriate programs for pilots.

In addition, using an FCM model to probe the human factors associated with startle resilience could help inform pilots training on optimal aeronautical decision-making during exigent circumstances or the prompt resolution of a fully developed airplane upset during flight operations. See [65,66,67,68] for a detailed treatment of aircraft upsets.

Furthermore, it has the added benefit of providing a roadmap for formulating experimentation protocols capable of instigating startle. The goal, as mentioned previously, is to provide valuable insights into startled responses that lead to and intensify performance degradation.

Undeniably, a startled individual is more prone to applying instinctive reactions, which might be inappropriate when process and precise knowledge are crucial to delivering a successful outcome.

### 3.2. Modeling Startle Causality with FCMs

The mapping process, as previously discussed, provides the basis for objective evaluation of any experimentation appropriateness and its efficacy, analysis of acquired data, and any computation efforts required to understand startle better. Additionally, the mapping process convergence plot outputs (depicted in the discussion section) provide a hierarchy to the crucial factors considered essential to understanding the startle process drivers in the GA context.

#### 3.2.1. Codification

For developing the FCM model for startle, four principles of practice, according to [47,51,69], are adopted to populate the map connections. These are as follows:

a. Choose the number N and kind of concepts *C_i_* of the FCM. In this case, we achieve this based on the HFACS framework to determine a total “N” of 19 human factors, with each element representing a concept C.
b. Determine the direction of connections and interactions amongst the concepts.
c. Use an inference rule to describe the relation between two concepts and infer a fuzzy linguistic set (weight) for the interconnection between the concepts.
d. Linguistic weights for every interconnection are combined and transformed into numerical weights.

After briefing on what constitutes a startle (within a piloting task), the expert(s) create a “fuzzy” correlation of the causal factors to the probability of being startled. This judgment process was done by providing weightings based on a linguistic representation of causal variables, by ascribing levels of perceived truth in the continuum [0, 1]. The implication is that an FCM linking those weightings enables the researcher to capture stakeholders’ perception of a system (or problem) structurally and build intuitive system representations from these subsequently. Such a rapid prototyping abstraction is beneficial for time-pressed incident management situations, fault finding, and decision-making efforts, as evidenced in [50,70,71]. Next, a questionnaire was administered to eight domain experts consisting of pilots and non-pilots. The experts, in this case, were selected for this proof of concept based on opportunity sampling for convenience. An essential part of choosing these experts is that they have significant knowledge of aviation and safety-critical systems, with considerable decision-making exposure.

The experts then had a week to consider the listed items before responding. Coincidentally, given the researcher’s active involvement in the aviation industry, participants were easily acquired for the survey. Appendix A shows the questionnaire used to survey the experts for the interested reader. The HFACS framework distils into 19 causal concepts deemed relevant to the susceptibility of a pilot to startle. This susceptibility to startle is notionally considered a degraded form of situation awareness that diminishes visual and operational comprehension of an ongoing situation. To manage the problem space dimensionality, the top 12 causal factors from ordering the experts’ responses are decision-driving inputs for the mapping algorithm to the end of inducing a startle response.

Note that the network algorithms are discussed extensively in [54,72,73]. The concept nodes naturally need to be primed with initial weights, and this occurs through a rule-based fuzzy inference system populated with domain expert judgment. These knowledge-based aggregated weightings provide a guided understanding of the possible correlations between the human factor concepts during the execution of a high cognitive workload. This understanding is crucial to deciphering how decision errors may be alleviated when the pilot is startled. Moreover, this supposition paves the way to establishing an experimental framework for investigating the problem as a specific case study. In this regard, the FCM convergence helps to establish the concept (node) hierarchy, of human factors interactions, for a structured analysis of how startle may propagate given an unexpected high-stress event.

The following representation is adopted from the work of [74] using the VIKOR technique. It introduces the definitions of a triangular fuzzy number concept used for linguistic variable associations (Table 2) based on items formulated on the FCM human factor concepts. The triangulation of fuzzy numbers complements the previously discussed definitions of fuzzy variable values, facilitating an objective sliding-scale boundary where the experts’ opinions for each concept consideration sit (see Table 2 for rating scales).

The triangulation concept represents a fuzzy piecewise continuous number, A, denoted by (*a*_1_, *a*_2_, *a*_3_) with a membership function ($\mu_{\tilde{A}}\left(x\right))$ defined as:

$$\mu_{\tilde{A}}\left(x\right)=\{\begin{array}{c} \frac{x-a_{1}}{a_{2}-a_{1}},\;\;for\;a_{1}\leq x\leq a_{2}\\ \frac{a_{3}-x}{a_{3}-a_{2}},\;\;for\;a_{2}\leq x\leq a_{3}\\ 0,\;\;elsewhere \end{array}$$

The linguistic rating used to judge the influence of startle causal factors can thus be represented by a value that is not a crisp number. Such representation of terminology used to guide each expert’s judgment when responding to questionnaire items is associated with natural language to avoid any possible ambiguity. This linguistic representation considers the fuzzy nature of decision-making (particularly when an intangible, such as startle causality, is concerned) and provides a moderated association of values to each HFACS concept. Table 3 provides a summary of the expert demographic consulted for this study.

#### 3.2.2. Association

The FCM Expert Software tool [42] facilitates the creation of an associative map of the causal concepts (a recurrent neural network) based on the participating experts’ responses to the questionnaire items. The parameters selected to model the interaction between the concepts, include Kosko’s activation function rule with self-memory and the Sigmoid Transfer function [51,54,73]. The Kosko mathematical representation of FCMs, according to [54], assumes the following form:

*A_i_*^(*k*+1)^ = *f* (*A_i_*^(*k*)^ ∗ Σ *Aj*^(*k*)^
*W_ji_*) *for j* = 1…. N (4)

where *A_i_^(k)^* is the activation value of the concept, *f*(*x*) is a threshold (activation) function, and *j*! = *i* through each iteration of the model state values of the related concepts. The equation calculates concepts’ numeric importance in the FCM with specific nodes set up as static non-decision driving concepts in the map. In the case presented, concepts 12 through 19, bar 16, are static. A Sigmoid threshold transfer function regulates values associated with the concepts in the range [0, 1], and its mathematical equation is:

*f* (*x*) = 1/(1 + *e*^−*λ*. *x*^) (5)

where *λ* is a real positive number that determines the degree of fuzzification and *x* is the value *A_i_*^(*k*)^ at the equilibrium point. In this construct, concepts are activated by making its vector element 0 or 1, or in the range [0, 1].

The threshold function, when applied, reduces the unbounded weighted sum to a predetermined range. It facilitates qualitative comparisons between HFACS concepts from the fuzzy linguistic associations used in the graph. The subsequent inference process consists of computing the current state vector through time, for a fixed initial condition, with a successive substitution method [42,73,75] to compute any new state vectors showing the effect of the activated concept. The computation occurs by iteratively multiplying the previous state vector using standard matrix multiplication by the relational matrix:

*A*^(*k*)^ = *A*^(*k*−1)^ + (*A*^(*k*−1)^. *W*_(*k*)_) (6)

The iteration stops when a limit vector is reached, i.e., when *A*^(*k*)^ = *A*^(*k*−1)^ or when |*A*^(*k*)^ − *A*^(*k*−1)^| ≤ e, where e is a residual that describes the minimum error difference among the concepts, whose value depends on the application (and, in most applications, is equal to 0.001) [51,54]. Thus, a final vector *A_f_* materializes, where scenario concepts clarify the specific decision flow of that iteration. Essentially, the network automatically finds any relationships in the input data and subsequently translates any discovered relationships into outputs, a form of unsupervised learning with no training dataset, implying an absence of feedback from the network environment/system.

The literature has three main threshold functions: Bivalent, Trivalent, and the Logistic Signal, a case known as the Sigmoid function [42,72,75,76]. The FCM expert tool used for this present work applies the sigmoid function. It seems to have the edge over the others, especially where vision system performances and eye tracking are concerned [75,77]. Therefore, the Bivalent and Trivalent options are restrictive and not considered appropriate in this study.

Modification of the weight matrix of the mapped concepts for what-if analysis is possible using well-established learning algorithms [47,51,54,69,71]. According to [54,78], three main approaches for handling the task of FCM training have emerged. These include Hebbian (signal, competitive, differential, or differential competitive), evolutionary, and a hybrid (of the two previously mentioned) type of machine learning algorithm. Extant literature on these algorithms is extensive, and they are outside the scope of this paper. Practically, the mechanism of the network state for the mapping matrix is updated at each time step. The update ensues by using a modified current state vector sequencing. The weights *W_ij_* value of the edge linking concepts *C_i_* and *C_j_* propagate by a discrete version of the differential Hebbian law. The activation Hebbian learning (AHL) process, which this represents, provides a procedure where the weight matrix of the FCM, through time steps, is modified to model the system’s behavior iteratively. Mathematically, this discrete version assumes the form:

*W_ij_* (*t* + 1) = *W_ij_* (*t*) + *µ_t_* (∆*C_i_* (*t*). ∆*C_j_* (*t*) − *W_ij_* (*t*)) (7)

where ∆*C_i_* is the change in the *i*th concept through consecutive states and:

∆*C_i_* (*t*) = *C_i_* (*t*) − *C_j_* (*t* − 1) (8)

The learning coefficient *µ_t_* gradually decreases over time, based on the following equation:

*µ_t_* = 0.1[1 − (*t*/(1. 1*N*)] (9)

The constant *N* is chosen to ensure *µ_t_* remains positive always. This value of *N* is equal to the number of observed state iterations. As there is no consideration of the time relationship between the concepts in the FCM, the model connotes a general representation of the scenario or system. Such heuristic methods reasonably estimate near-optimal output values with a pragmatic optimization of the error function. Table 4 provides an overview of these concepts and presents them within their respective themes within the HFACS framework.

In the final FCM model built for this research (as depicted earlier in Figure 3), there are 20 nodes, where C20 is the target node (e.g., the startle node). The red nodes are influential nodes on the target node while the blue nodes are not. The weights associated with the edges depict the degree of influence on the target concept. The blue causal factors in the map are all independent variables, much like the red factors, and they all feed into the overall picture of human factors that can lead to the startle node. However, in terms of their contribution to the convergence maps, they are not set to be decision drivers. Therefore, only the red items have a decisive impact on the outcomes.

We can deploy the fuzzy model to simulate, test, and objectively analyze parameters’ influence for system behavior prediction. Such accessibility is invaluable to help develop appropriate training protocols required to improve pilots’ outcomes. Table 5 provides an overview of these concepts and presents them in a ranked order as chosen by a panel of experts. For robustness, the randomized expert inputs reflect a mixed-effects model of identified variables from the HFACS framework. This process helps to minimize uncontrollable domain noise and ensures an objective assessment of what level or degree of truth these independent causal variables hold.

## 4. Discussion

### FCM Results

**Figure 4:**
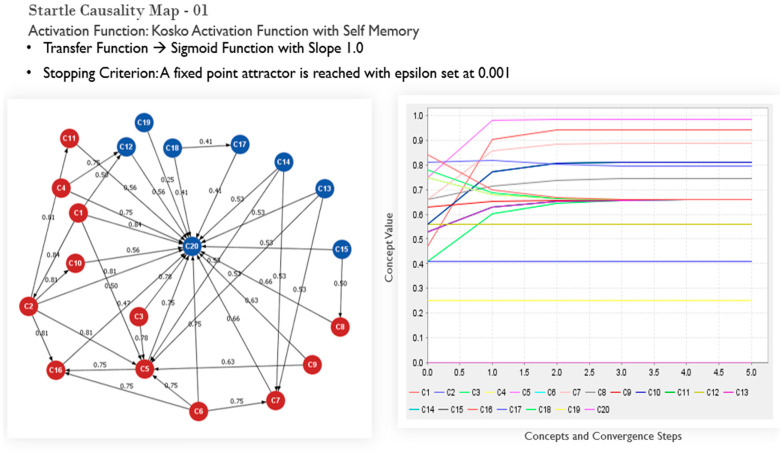
Initial mapping test output with associated convergence output plot: the *x*-axis shows the steps to convergence for a particular iteration; the y-axis shows the converged value of concepts contributing to the startle output.

**Figure 5:**
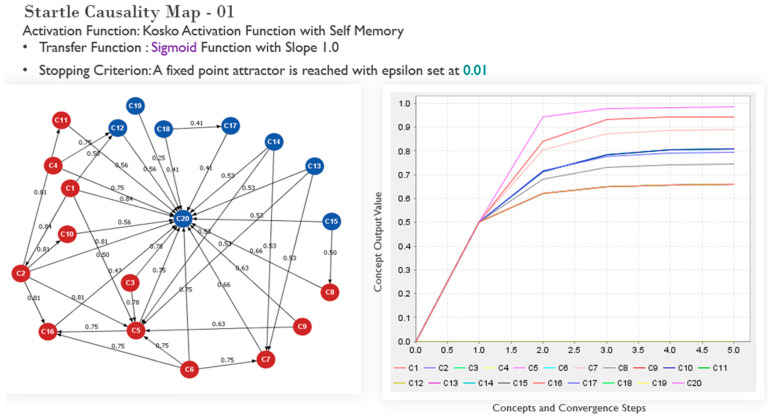
Initial mapping and associated convergence output plot with the stopping criterion adjusted to test the output behavior.

**Figure 6:**
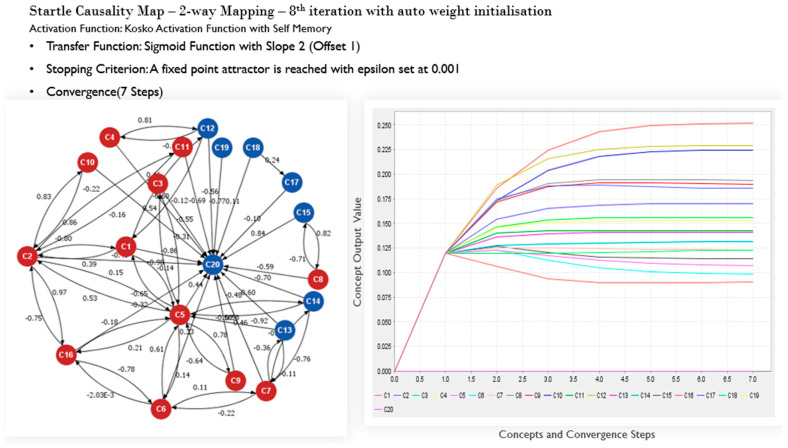
Startle causality map and associated convergence output: showing concepts mapped in both directions, and with initial concepts weights autogenerated through iterations (NOTE: The 8th iteration is shown. Items with 2-way mapping are as per Table 6).

The graphs depicted in Figure 4, Figure 5 and Figure 6 illustrate the efficacy of fuzzy cognitive maps for interrogating “what-if” scenarios, following the experts’ input for the initial weightings of the causal concepts. The numbers describe the associative relationships between the human factors-related concepts and the startled state of mind. In the maps depicted, the startle node and other nodes in blue do not carry a decision driving function for the final output value representing influence. Semantically, the maps depict the relatedness of human factor concepts. It visualizes a mental model about the relationship between the domain concepts when first created. Even a first iteration of the map can provide direction for contemplating training or experimentation protocols however, this would not be robust enough and no devoid of subjectivity. To mitigate this limitation, further interaction amongst concepts is captured in subsequent iterations of the map. Finally, the mapping outputs a convergence plot that ascribes a numerically weighted hierarchy to the human causal factors, determined through an inference algorithm based on population heuristic search methods [51,73,79]. The outputs of the maps (convergence plots) provide a ranking of the causal factors according to their calculated propensity as a root cause for a pilot’s in-flight startled reaction. For the efficacy of analysis, the top four concepts, following each mapping process iteration, are considered as a basis for conceiving training designed to build resilience to startle in pilots. In Figure 4, the outputs converge to a top-four causal factor hierarchy of concepts 5, 9, 7, and 2 (in descending order of criticality as contributing factors to startle). Table 5 shows these concepts to be poor appraisal of the situation, poor visual references due to weather, poor communication skills (such as with air traffic control), and an unskilled pilot.

These concepts are plausible factors for GA pilots (of interest to the current study) but could also be the case for experienced pilots. This example demonstrates the power of the FCM to enable rapid, iterative, and objective consideration of causal factors for an intangible output concept, such as startle. However, these results only prove the utility of the simulated initial maps and yield some interesting results. Running the iteration, a second time for Figure 5 where epsilon is adjusted, the hierarchy of concepts converge to concepts 5, 1, 7 and 2. Concept 1 (Insufficient Training/Lack of Concurrency) being the difference between the outputs. The convergence map output visualisation is offset to

Figure 6 shows a map built with a two-way connection between concepts, as highlighted in Table 6. Startle, in this case, is also considered to be static. In this example, the top four driving factors of startle are now C16 (lack of assertiveness), C12 (poor ADM knowledge), C10 (poor preparation), and C8 (stress) The auto-initialization of subsequent weights through iterations, evidences the FCM’s capability to remove subjectivity in the process of developing an understanding of the causal mechanisms of in-flight startle.

The post-convergence concept hierarchies (such as the four we highlight above) can be referenced in the embodiment and delivery of flight simulation training protocols to test the influence of any one of these causal agents.

It is essential to highlight some semantic understanding of how the FCM concepts are mapped, as shown in Table 6. We use the example of mapping ADM to faulty/uncalibrated instruments as a pertinent example for this study, as this relationship involves the pilot’s visual processing of instrument readings and the possible ensuing decisions (actions) the pilot might take in an emergency. We consider the scenarios of interest where the pilot’s decisions and behaviors are primarily directed by what can be experienced and cognitively processed inside the cockpit. Therefore, ADM is mapped to an instrument that is faulty/uncalibrated on the premise that ADM starts from the pre-flight planning and onboard check phases through to landing and other aspects in between, associated with a particular flight [55,80]. For example, we consider a situation involving a fledgling pilot who inadvertently gets into an unexpected separation incident because s(he) failed to adjust the instrumentation for the local altitude above sea level before take-off, thus leading to false readings from the instruments (altimeter in this case).

The following tables outline the outputs of the mappings and provide inferences on the impact of human factors driving startle. They also offer a visual assessment of the general behavior of the models.

Table 7 shows the iterations and outputs of the initially created mapping (Figure 4 and Figure 5) according to Table 6 but in one direction. In this case, as in all other tested iterations, startle is not set as a decision concept and is considered as receiving inputs in the context of other interactions amongst map nodes. In addition, adjusting the fixed-point attractor (i.e., selecting epsilon to 0.001, instead of 0.01) caused no fundamental differences to emerge in the model’s performance and output compared to the baseline test map. However, it is also helpful to note that with the adjustment of epsilon, concepts C5 (poor situation appraisal), C1 (insufficient training), C7 (poor communication with ATC), and C2 (unskilled pilot) emerge as the top four dominant factors in this scenario. Again, these outputs are like the first iteration except for the concept C1, insufficient training, which does not appear when the fixed-point attractor was 0.01.

Table 8 shows a short experiment with the 2-way mapping (Figure 6) but without autogenerated initial weights through iterations. These outputs, as previously discussed, highlight how the FCM facilitates the rapid exploration of the connections between these human factor concepts and poor performance, should a startling event occur.

The outputs of Table 9 are preferred because the iterations provide results based on randomization of the concept weights in the map from the second iteration onward. In addition, the auto-randomization of the concept weights adds a layer of robustness and a high level of objectivity since the experts have not provided input into the subsequent initialized weightings.

Based on the outputs of questionnaires, potential performance impact routes are charted, using the FCM of human factor concepts. The concepts used in the mapping are chosen based on the inference of the predominance of visual perception, visual attention, task management, decision-making, and memory mechanisms abstracted from the HFACS and aligned with the SEEV framework as discussed in Section 2.

As shown in the literature, in critical decision-making situations (e.g., an inflight emergency), human factors, such as automation bias and inexperience, could force a pilot to maintain a heavy reliance on cues (typically visual) originating from potentially failing sources of information in the cockpit. Crucially, the pilot’s perception of other environmental elements may also be significantly eroded by the emergence of an unforeseen event, further adding complexity to the problem [2,14]. Ultimately, this could lead to poor aeronautical decision-making (ADM). In such an evolving situation, the startled pilot may exhibit an instinctive reactionary behavior with a strong tendency towards subsequent mishaps [14,22].

The FCM framework affords a systematic approach for codifying the relationships amongst human factors and their potential for driving a pilot to startle in a dynamically evolving emergency. For this study, the FCM implementation provides objective insight into the startled mind through a quasi-Delphi questionnaire and analysis process involving a cohort of aerospace and aviation experts. Distilling relevant key concepts, the fuzzy mapping process is developed to represent an intuitive view of the problem space. In this case, we proceed to consider the possibility of physiological (e.g., eye-tracking) information, representative of visual acuity (conceived as a correct reading of instrument indicators, such as take-off speed), as a function of situation awareness and decision-making during an unexpected and potentially startling event.

## 5. Summary and Future Work

As mentioned, FCMs have been used to successfully model and evaluate processes characteristic of human interaction with complex systems to great effect [49,50,70]. The Salience, Effort, Expectancy and Value (SEEV) framework [27,44] also plays a central role in guiding the presented research. It facilitates a link between human factor modeling and a computational cognitive structure, representing human capabilities and limitations on allocating visual attention to cockpit resources [37,43,44].

To build the FCM model of startle predisposition driven by human factors, we provided a summary of the HFACS framework concerning piloting a GA aircraft, outlined as a total of 19 concepts: insufficient training/lack of concurrency (C1), unskilled pilot (not rated for aircraft type for instance) (C2), fatigue/tiredness (C3), faulty/uncalibrated instrument readings (C4), appraisal of an evolving situation (C5), medication/drugs (C6), communication (ATC) (C7), stress (C8), availability of visual references (C9), preparation (flight/route planning and pre-flight checks) (C10), resource awareness/crew resource management (CRM) (C11), lack of ADM knowledge (perceive—process—perform) (C12), distractions (phone call, in-flight conversations) (C13), cockpit ergonomics/information layout (C14), time pressures (C15), lack of assertiveness (C16), complacency (route familiarity) (C17), norms (C18);, and part 91 rules (less stringent rules) (C19). Note C, followed by a number, is the concept label for the FCM model. All these items are relevant for startle resilience but to varying degrees. As highlighted earlier, we limit the decision-making nodes to the top 12 in hierarchical order. Concepts are accounted for in the map because they all represent the pertinent aspects of the problem studied.

As shown in Table 9 earlier, the auto-generation of weight initialization within the map for eight iterations converges the problem space. We obtain a top-four critical concept as the drivers of a startled reaction, including concept 16 (a lack of assertiveness), concept 12 (poor ADM knowledge), concept 10 (poor preparation), and concept 2 and 14 (lack of skill/ergonomics). Interestingly, given the randomization of weights through each of the iterations, these top four items sit well as a plausible root cause for poor startle resilience given an abrupt activation of inactive frames during level 1 SA and subsequently, actioned by a fast appraisal response of the situation (see Figure 1). The contention here is that such “fast appraisal” at level 1 of situational awareness [20,21,81] could lead to an instinctive (“knee-jerk”) and a wrong application of flight control laws.

Given the possible combinations of the human factor candidates, the fuzzy mapping process helps target real-world mitigations that map to causal factors. In the case of in-flight startle, flight training developers can assess simulated remedial programs in a controlled, safe, and repeatable manner based on the priority items identified. Furthermore, the analysis of any such programs leads to a better understanding of how task performance may be affected.

Table 10 reflects the most frequently occurring (“mode”) concepts through the mapping iteration outputs to indicate the top four factors determined from the FCM simulations. The LB-A (simulations on 2-way concept mapping) results allow us to conclude that startle resilience in an unexpected situation is most affected by a lack of assertiveness (C16), poor ADM knowledge (C12), inadequate preparation (C10), and low skill (C2). (C14). Cockpit ergonomics come into play as a joint equally occurring item for causal concept 4, which could mean that there is room for improving this area as well. There are efforts ongoing in this domain, such as those highlighted in [29,82,83].

We conclude that startle causality FCMs considering human factors interactions serve two essential high-level functions: explanatory prototyping—what is happening in the system given human factors? and predictive—what will happen next and how can we prepare pilots better? Furthermore, as a reflective tool, the utility of fuzzy cognitive maps means researchers can readily adapt their maps to new knowledge in the domain of interest. Strategically, the FCM helps determine a clear line of action for enabling solution generations for the problem space under consideration. Moreover, as airspaces become increasingly populated, this has implications for human performance modeling and potential error mitigations. Finally, although this study seeks to understand startle causality for the fledgling GA pilot, this bodes well for guiding experimentation and analyses, even for experienced pilots in general.

In the future, we recommend experiments accounting for the FCM outputs in future work. As flight simulation continues to be a widely accessible and relatively inexpensive but vital part of pilot training [12,67,84,85], given the FCM findings, we can develop flight simulation experimental studies to test the dynamic process of task performance inflight, with unexpected situations embedded. Such a study could yield invaluable understanding from analyses of performance metrics, such as visual acuity or attention to areas of interest according to the SEEV model. Researchers can make such analyses based on physiological data captured from an eye tracker.

Over the last decade, eye-tracking has increasingly become a desirable proposition for enhancing pilot training according to trends in the aviation industry [29,68,86,87,88,89]. Following this trend, incorporating pupillometric analysis can improve the efficient review of pilots’ responses to potentially startling simulation exercises. Therefore, such an addition plays a part in the future of flight training for pilots in the loop, interacting with increasingly complex and automated systems, completing complex tasks as would be done in a cockpit.

As an affecting agent in performance loss in an age of accessibility to modern advances in ML, computing power, sensor sophistication (such as in eye trackers), and more, human limitations, such as startle, can be studied effectively in simulations aligned with an objective and robust hierarchy of causal factors determined by the fuzzy mapping process. Furthermore, given the rapid prototyping of initial weights for FCMs using modern software, a larger cohort of experts could be consulted through a Delphi process survey similar to those done in [49,74,90] to improve or validate the rankings in this study. Such outputs and any further improvements should help guide the development of simulated training that pilots can transfer to real-life operations.

## Figures and Tables

**Table 1:** “Fast appraisal” startle conceptualization for an unexpected “clear air turbulence” event ^1^.

Stages	Event Sequence	Description
Stage 1	Unexpected Stressor	Event occurrence to the active frame of mental operation (Thalamus)
Stage 2	Stimulus to the Amygdala	Appraisal of Events
Stage 3	Event intensity perception	Visual appraisal process (sympathetic nervous system and adrenal cortical systems are activated)
	“Fast Appraisal” pathway	Wrong/incomplete information about threat intensity is adopted
Stage 4	Suboptimal gaze pattern	Ineffective Visual Acuity (Collecting wrong/irrelevant information)
Stage 5	Global understanding degraded	Understanding of scenario is suboptimal/hampered
Stage 6	Fight/Flight reaction	The threat is confirmed, and the reaction is a “Knee Jerk” response
Stage 7	The pilot exhibits startled behavior	Poor ADM/LOC/Poor task scores in a simulator/Physiological outputs

^1^ The conceptualized process reflects the fast appraisal pathway in Figure 2 and aligned with ideas on the NDM and SEEV aspects of the problem space. Note that the sequence conceptualized can occur in a matter of milliseconds.

**Table 2:** Fuzzy linguistic ratings.

Linguistic Rating Terminology (Judgement of Influence)	Triangular Fuzzy Numbers (Numerical Rating of Factor’s Influence)
Very Low Influence	0.00, 0.00, 0.25
Low Influence	0.00, 0.25, 0.50
Medium	0.25, 0.50, 0.75
High Influence	0.50, 0.75, 1.00
Very High Influence	0.75, 1.00, 1.00

**Table 3:** Summary of experts.

Expert	Occupation
LC	Chief Engineer (Aerospace Safety Systems)
AR	Aerospace Design Engineer
MK	Aerospace Design Engineer
TH	Aerospace Manufacturing Engineer
AH	Ex-UK CAA Safety Expert
RM_PPL1	Aerospace Engineer & GA Pilot
SP_PPL2	Aerospace Engineer & GA Pilot
JS_PPL3	Aerospace Engineer & GA Pilot

**Table 4:** Rated startle drivers’ subgroupings based on HFACS concepts.

Concepts of Acts and Omissions	Concept Description	Rating
C6	Medication/Drugs	0.75
C10	Preparation (Pre-Flight Checks)	0.56
C11	Awareness (CRM)	0.56
C16	Lack of Assertiveness	0.47
C17	Complacency	0.41
**Concepts of Preconditions & Local Factors**	**Concept Descriptions**	**Rating**
C2	Unskilled Pilot	0.81
C4	Faulty/Uncalibrated Instruments	0.75
C9	Visual References	0.63
C15	Time Pressures	0.53
C14	Cockpit Ergonomics (Information Layout)	0.53
C13	Distraction (Inflight)	0.53
**Concepts of Supervision and** **Local Management**	**Concept Descriptions**	**Rating**
C5	Poor Situation Appraisal	0.75
C7	Poor Communication (ATC)	0.66
C12	Lack of ADM Knowledge/Training	0.56
**Concepts of Organizational Influences**	**Concept Descriptions**	**Rating**
C1	Insufficient Training	0.84
C3	Fatigue/Tiredness	0.78
C8	Stress	0.66
C18	Norms (Familiarity)	0.41
C19	Part 91 Rules	0.25

**Table 5:** HFACS variables aggregated and sorted in ranking as determined by aerospace and aviation experts.

Concepts	Causal Factors (Independent Variables)	LC	AR	MK	TH	AH	RM_ PPL1	SP_ PPL2	JS_ PPL3	Ranked Mean
C1	Insufficient Training/Lack of Concurrency	0.75	0.75	0.75	0.75	1.00	1.00	1.00	0.75	0.84
C2	Unskilled Pilot (Not rated for Aircraft Type for instance)	0.75	1.00	0.75	0.75	0.75	1.00	1.00	0.50	0.81
C3	Fatigue/Tiredness	0.50	0.75	0.50	1.00	1.00	1.00	0.75	0.75	0.78
C4	Faulty/Uncalibrated Instrument Readings	1.00	0.75	0.75	1.00	1.00	1.00	0.25	0.25	0.75
C5	Appraisal of Evolving Situation	0.75	1.00	0.75	1.00	1.00	0.50	0.50	0.50	0.75
C6	Medication/Drugs	1.00	0.75	0.50	0.25	1.00	1.00	0.50	1.00	0.75
C7	Communication (ATC)	1.00	0.75	1.00	0.50	1.00	0.50	0.25	0.25	0.66
C8	Stress	0.50	0.75	0.75	0.50	0.75	0.75	0.75	0.50	0.66
C9	Availability of Visual References	0.75	0.75	0.50	0.75	0.75	0.75	0.25	0.50	0.63
C10	Preparation (Flight/Route Planning, Pre-Flight checks)	1.00	0.25	0.50	1.00	1.00	0.25	0.25	0.25	0.56
C11	Resource Awareness/Crew Resource Management (CRM)	0.25	0.50	0.25	0.75	1.00	0.75	0.25	0.75	0.56
C12	Lack of ADM knowledge (Perceive–Process–Perform)	0.25	0.50	0.25	0.50	1.00	0.50	0.75	0.75	0.56
C13	Distractions (Phone Call, In-Flight Conversations)	0.75	0.50	0.5	0.25	1.00	0.50	0.5	0.25	0.53
C14	Cockpit Ergonomics/Information Layout	0.50	0.50	0.25	0.75	1.00	0.25	0.25	0.75	0.53
C15	Time Pressures	0.50	0.50	0.25	0.25	1.00	0.50	0.50	0.75	0.53
C16	Lack of Assertiveness	0.50	0.75	0.25	0.25	1.00	0.50	0.25	0.25	0.47
C17	Complacency (Route Familiarity)	0.75	0.50	0.00	0.25	1.00	0.50	0.25	0.00	0.41
C18	Norms	0.25	0.50	0.25	0.25	1.00	0.25	0.25	0.50	0.41
C19	Part 91 Rules (Less Stringent Rules)	0.50	0.00	0.25	0.00	0.50	0.25	0.50	0.00	0.25

**Table 6:** Mapping of the concepts in the FCM model ^1^.

Mapping	Description
1 < > 12	Insufficient Training—Lack of ADM
1 < > 5	Insufficient Training—Poor Situation Appraisal
1 < > 2	Insufficient Training—Unskilled Pilot
6 < > 5	Medication/Drugs—Poor Situation Appraisal
6 < > 7	Medication/Drugs—Poor Communication (ATC/Other Aircraft)
6 < > 16	Medication/Drugs—Lack of Assertiveness during emergency
2 < > 10	Unskilled Pilot—Poor Preparation (Pre-Flight Checks)
2 < > 16	Unskilled Pilot—Lack of Assertiveness
2 < > 11	Unskilled Pilot—Poor Crew Resource Management (CRM)
2 < > 5	Unskilled Pilot—Poor Situation Appraisal
5 < > 16	Poor Situation Appraisal—Lack of Assertiveness
15 < > 8	Time Pressures—Stress
9 < > 5	Visual References (Unavailable due to weather)—Poor Situation Appraisal
4 < > 12	Faulty/Uncalibrated Instruments—Poor ADM
13 < > 7	Distractions—Poor Communication (with ATC/Other Aircraft)
3 < > 5	Fatigue/Tiredness—Poor Situation Appraisal
14 < > 5	Cockpit Ergonomics (Usability and effectiveness)—Poor Situation Appraisal
14 < > 7	Cockpit Ergonomics—Poor Communication

^1^ Double arrow represent 2-way mapping between concepts in Figure 6. Semantically, the items show the relatedness of concepts in the human factor context. A mental model with the utility of providing a basis on which we can contemplate experiments.

**Table 7:** FCM test runs—map 1.

FCM	Activation Function	Slope	Offset	Epsilon	Steps	1st	2nd	3rd	4th
Test1	Sigmoid	1.0	1.0	0.001	5	5	9	7	2
Test2	Sigmoid	1.0	1.0	0.010	5	5	1	7	2
Test3	Sigmoid	1.0	0.3	0.001	8	5	2	1	16
Test4	Sigmoid	4.5	0.5	0.001	8	5	1	10	7
Test5	Sigmoid	5.5	0.5	0.001	22	7	15	1	10

**Table 8:** Mapping test with a 2-way connection (LB) between concepts and no autogenerated weights.

FCM	Activation Function	Slope	Offset	Epsilon	Steps	1st	2nd	3rd	4th
LBTest1	Sigmoid	1	1	0.01	8	2	1	16	6
LBTest2	Sigmoid	1	1	0.10	8	7	1	16	6
LBTest3	Sigmoid	2	1	0.10	17	2	2	16	6

**Table 9:** Map outputs from autogenerated weight mapping.

FCM	Activation	Slope	Offset	Epsilon	Steps	1st	2nd	3rd	4th
LB-A-001	Sigmoid	2	1	0.001	8	2	6	1	9
LB-A-002	Sigmoid	2	1	0.001	7	9	5	3	1
LB-A-003	Sigmoid	2	1	0.001	8	6	1	10	2
LB-A-004	Sigmoid	2	1	0.001	7	12	2	13	14
LB-A-005	Sigmoid	2	1	0.001	12	16	12	9	2
LB-A-006	Sigmoid	2	1	0.001	9	1	13	15	14
*LB-A-007 (Chaotic) ^1^	Sigmoid	2	1	0.001	100	7	1	9	3
LB-A-008	Sigmoid	2	1	0.001	7	16	12	10	8

^1^ The 7th iteration of this map is not used for conclusions as it took too long to converge (100 steps) in comparison to other iterations signifying some instability in the model. Epsilon is maintained at 0.001.

**Table 10:** Mode of FCM convergence output values ^1^.

FCM	Steps to Convergence	1st	2nd	3rd	4th
Test Output (Mode)	5	5	1	7	2
LB Test Output (Mode)	8	2	1	16	6
LB-A (Mode)	8	16	12	10	14/2

^1^ LB-A meaning “Linked Back (2-way mapping) with Auto weight generation”.

## Data Availability

All pertinent data for this article have been reported in the body of this submission.

## Abbreviations

ADM	Aeronautical Decision Making
AHPLS	Attention-related human performance-limiting states
AIAA	American Institute of Aeronautics and Astronautics
AOI	Area of Interest
CAT	Clear Air Turbulence
CF	Causal Factors
ESD	Event Sequence Diagrams
FAA	Federal Aviation Authority
FCM	Fuzzy Cognitive Map
GA	General Aviation
HFACS	Human Factors Analysis and Classification System
ICAO	International Civil Aviation Organisation
ICATEE	International Committee for Aviation Training in Extended Envelopes
LOC	Loss of control
MIDAS	Man-machine Integration Design and Analysis System
NDM	Naturalistic Decision-Making
SA	Situational awareness
SEEV	Salience, Effort, Expectancy, Value

## Appendix A. Fuzzy Cognitive Mapping (FCM) Questionnaire

### Appendix A.1. Background

“*In aviation, the startle effect can be defined as an uncontrollable, automatic reflex that is elicited by exposure to a sudden, intense event that violates a pilot’s expectations*” [22]. Given the above definition, kindly rank the following items (abstracted from the HFACS Framework) in the table based on your understanding/opinion of their effect on startling event readiness, ergo their potential to impact efficient task execution by pilots in the General Aviation (Part 91) Operations. Using Table from Appendix A.3, Specifically, consider the potential for a loss of control (LOC) due to the causal factors following such a startling event.

### Appendix A.2. Causal Factors

**Startle—LOC Causal Factors**	**Ranking (0–1)**
Part 91 Rules (Potentially Less Stringent Rules)	
Pre-Flight Checks	
Faulty/Uncalibrated Instrument Readings	
Preparation (Flight/Route Planning Etc.)	
Lack of Visual References	
Appraisal of Evolving Situation	
Fatigue/Tiredness	
Insufficient Training/ Lack of Concurrency	
Unskilled Pilot	
Cockpit Ergonomics/Information Layout	
Resource Awareness/CRM	
Stress	
Communication (ATC)	
Distractions (Phone Call, In-Flight Conversations)	
Complacency (Route Familiarity)	
Time Pressures	
Lack of ADM Knowledge (Perceive—Process—Perform)	
Lack of Assertiveness	
Norms	
Medication/Drugs	

### Appendix A.3. Causal Factors Linguistic Ratings

The following table provides a linguistic terminology rating for your convenience. Please select from the associated numerical rating triangulation set. These numbers represent your judgement of the factor’s influence on performance in the event of a startling incident, with a potential for LOC.

**Linguistic Rating Terminology** **(Judgement of Influence)**	**Triangular Fuzzy Numbers** **(Numerical Rating of Factor)**
Very Low Influence	0, 0, 0.25
Low Influence	0, 0.25, 0.50
Medium	0.25, 0.50, 0.75
High Influence	0.50, 0.75, 1.00
Very High Influence	0.75, 1.00, 1.00

### Appendix A.4. Demographic Information

1. Do you have any Federal Aviation Administration (FAA)/UK Civil Aviation Authority UK CAA pilot certifications or ratings?
2. If yes, what is the highest level of certificate that you hold?
  ○ Student Pilot
  ○ Sport Pilot
  ○ Recreational Pilot
  ○ Private Pilot
  ○ Commercial Pilot
  ○ Airline Transport Pilot (ATP)
  ○ Other (Please spec*):
3. What category or categories of aircraft are on this highest certificate? If applicable, mark all the following that apply.
  ○ Airplane
  ○ Rotorcraft
  ○ Glider
  ○ Lighter Than Air
  ○ Powered lift
  ○ Powered Parachute
  ○ Weight Shift
4. What class or classes of aircraft are on this highest certificate? If applicable, mark all the following that apply.
  ○ Single Engine Land
  ○ Single Engine Sea
  ○ Multi-Engine Land
  ○ Multi-Engine Sea
  ○ Balloon Airship
  ○ Helicopter
  ○ Gyroplane
  ○ Other (Please specify):
5. Do you have an instrument rating? Please indicate as necessary
  ○ No
  ○ Yes
6. Please indicate any non-pilot Federal Aviation Administration (FAA)/ UK CAA certifications that you hold:
  ○ None
  ○ Flight Engineer
  ○ Flight Navigator
  ○ Air Traffic Control Specialist, Control Tower Operator or Equivalent
  ○ Aircraft Dispatcher
  ○ Mechanic, Avionics Technician, Repairman, or Equivalent
  ○ Other (Please specify):
7. What is your gender?
  ○ Female
  ○ Male
  ○ Prefer not to answer
8. What is your current Age?
  ○ ___Age
  ○ Prefer not to answer
